# Assessment of diagnostic value of unilateral systematic biopsy combined with targeted biopsy in detecting clinically significant prostate cancer

**DOI:** 10.1515/med-2024-1048

**Published:** 2024-10-04

**Authors:** Jian Wu, Guang Xu, Lihua Xiang, Lehang Guo, Shuai Wang, Lin Dong, Liping Sun

**Affiliations:** Department of Medical Ultrasound, Center of Minimally Invasive Treatment for Tumor, Shanghai Tenth People’s Hospital, Ultrasound Research and Education Institute, Clinical Research Center for Interventional Medicine, School of Medicine, Tongji University, Shanghai, 200072, China; Department of Medical Ultrasound, Center of Minimally Invasive Treatment for Tumor, Shanghai Tenth People’s Hospital, Ultrasound Research and Education Institute, Clinical Research Center for Interventional Medicine, School of Medicine, Tongji University, No. 301, Yanchang Middle Road, Jing'an District, Shanghai, 200072, China

**Keywords:** prostate cancer, targeted biopsy, diagnostic accuracy, sensitivity, unilateral systematic biopsy

## Abstract

**Objectives:**

This retrospective study assessed the diagnostic accuracy of targeted biopsy (TB) and unilateral systematic biopsy in detecting clinically significant prostate cancer (csPCa) in 222 men with single magnetic resonance imaging (MRI) lesions (Prostate Imaging Reporting and Data System [PI-RADS] ≥ 3).

**Methods:**

Patients underwent multiparametric MRI and MRI/ultrasound fusion TB and 12-needle standard biopsy (SB) from September 2016 to June 2021. The study compared the diagnostic performance of TB + iSB (ipsilateral), TB + contralateral system biopsy (cSB) (contralateral), and TB alone for csPCa using the *χ* ^2^ test and analysis of variance.

**Results:**

Among 126 patients with csPCa (ISUP ≥ 2), detection rates for TB + iSB, TB + cSB, and TB were 100, 98.90, and 100% for lesions, respectively. TB + iSB showed the highest sensitivity and negative predictive value. No significant differences in accuracy were found between TB + iSB and the gold standard for type 3 lesions (*P* = 1). For types 4–5, detection accuracy was comparable across methods (*P* = 0.314, *P* = 0.314, *P* = 0.153). TB had the highest positive needle count rate, with TB + iSB being second for type 3 lesions (4.08% vs 6.57%, *P* = 0.127).

**Conclusion:**

TB + iSB improved csPCa detection rates and reduced biopsy numbers, making it a viable alternative to TB + SB for single MRI lesions.

## 1 Introduction

Transrectal ultrasound-guided prostate biopsy is regarded as the gold standard procedure for diagnosing prostate cancer. The pathological findings from the biopsy are of paramount importance for accurately assessing the risk level of the tumor and determining the most appropriate treatment. It is recommended that biopsy techniques include both targeted and systematic sampling [1]. Although combined biopsies are advantageous for detecting more clinically significant prostate cancer (csPCa), the increased number of core punctures can result in elevated psychological distress and postoperative complications for patients [2].

Magnetic resonance imaging (MRI) has been demonstrated to have high sensitivity for the detection of prostate cancer and a notable negative predictive value [3]. It is particularly efficacious in identifying lesions categorized as Prostate Imaging Reporting and Data System (PI-RADS) score of 3–5. Furthermore, MRI-guided targeted biopsies have been demonstrated to identify a considerably higher proportion of csPCa compared to conventional standard biopsy (SB) [4]. Nevertheless, targeted biopsy (TB) may still be inadequate for detecting some cases of csPCa, as evidenced by SB identifying approximately 5% of missed cases [5]. The variability in the detection efficiency of cancer around the target site underscores the limitations of SB in accurately diagnosing prostate cancer. Bryk and colleagues conducted a study to quantify unilateral prostate lesions and observed that a systemic puncture on the target side resulted in a modest increase in the detection of significant cancers, as well as a modest increase in the detection of non-significant cancers. Conversely, a contralateral system paracentesis primarily increased the detection of non-significant cancers [6]. These findings imply that cancers not identified by TB are more likely to be detected through ipsilateral systemic biopsy (iSB).

The detection rate of csPCa has been demonstrated to fluctuate in accordance with the PI-RADS score. Studies have indicated that the detection rates for csPCa with PI-RADS scores of 3–5 are approximately 12–21, 39–48, and 72–73% [7,8], respectively. Moreover, higher PI-RADS classifications are associated with higher detection rates of csPCa. Prospective trials with a PRECISION design have confirmed the efficacy of utilizing fusion biopsies exclusively in patients with intermediate- to high-risk initial biopsies [9].

The diagnostic efficacy of TB in conjunction with iSB in comparison with combined biopsy remains uncertain, as does the potential for variation in risk stratification outcomes. In consideration of the multifocal nature of prostate cancer, unilateral lesions identified on MRI were selected as the focus of this study, with the objective of minimizing the impact of adjacent lesions. This approach enables a more accurate assessment of the impact of iSB and cSB on csPCA detection in cases with or without visible lesions.

**Figure 1:**
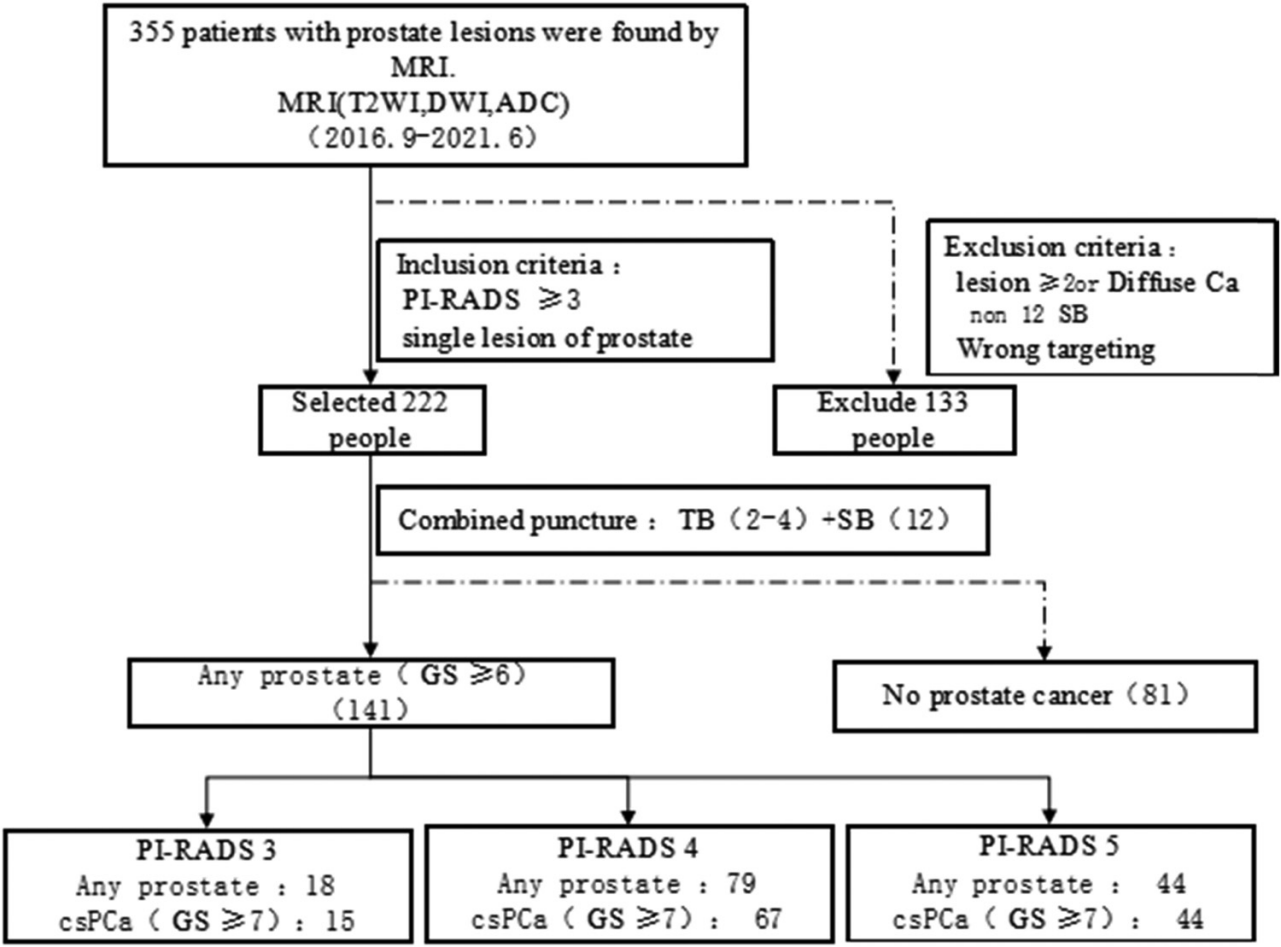
Inclusion and exclusion processes.

The aim of this study is to conduct a retrospective evaluation of the diagnostic effectiveness of TB in single lesions identified by MRI, both as a standalone procedure and in combination with iSB, in comparison with the combination of TB and SB (Figure 1).

## 2 Materials and methods

This retrospective study was approved by the Ethics Committee of Tongji University Affiliated Shanghai Tenth People’s Hospital (Shanghai, China; Approval No. SHSY-IEC-4.0), and each patient provided oral informed consent. Between September 2016 and June 2021, a total of 355 patients underwent multiparametric magnetic resonance imaging (mpMRI) examination as a result of elevated prostate specific antigen (PSA) (>4 ng/mL), abnormal rectal digital examination, or suspicious prostate lesions detected by ultrasound. All patients received positive mpMRI results (PI-RADS ≥ 3 points V2.0) and subsequently underwent MRI/ultrasound (US) fusion TB combined with 12-needle systematic biopsy (TB + SB). Inclusion criteria included the presence of a unilateral MRI finding. The MRI revealed the presence of unilateral lesions of the prostate, accompanied by clear pathological findings from the biopsy. Patients were excluded from the study if they lacked pathological confirmation of diffuse or multifocal prostate cancer, had not undergone a 12-core systematic biopsy, or exhibited evident fusion errors in TB. The lesion was identified as being located on the median line of the prostate. A total of 222 patients were included in this retrospective, self-controlled study.

### 2.1 mpMRI imaging

Two imaging devices and parameters were employed for mpMRI using a 3.0-T Discovery MR system equipped with surface-phased array coils (Verio, Siemens, Germany and Ingenia, Philips, Netherlands). The mpMRI protocol comprised T2-weighted imaging, diffusion weighted imaging (DCI), and dynamic contrast enhanced imaging (DCE). in accordance with the PI-RADS 2.0 guidelines. The two mpMRI scans were jointly interpreted by two experienced radiologists, Wang Shuai (with 3 years of experience) and Zhao Bing Hui (with over 7 years of experience in utilizing PI-RADS for prostate MRI interpretation).

### 2.2 Fusion imaging and biopsy

Fusion imaging and biopsy procedures were conducted using the MyLab Twice US scanner (Esaote, Genoa, Italy) and the Mindray Resona 9 ultrasound diagnostic instrument (Mindray, Shenzhen), both of which were equipped with a fusion software package, a 6–9 MHz biplane rectal transducer (TRT33; Esaote), and a real-time sensor navigation system.

**Figure 2:**
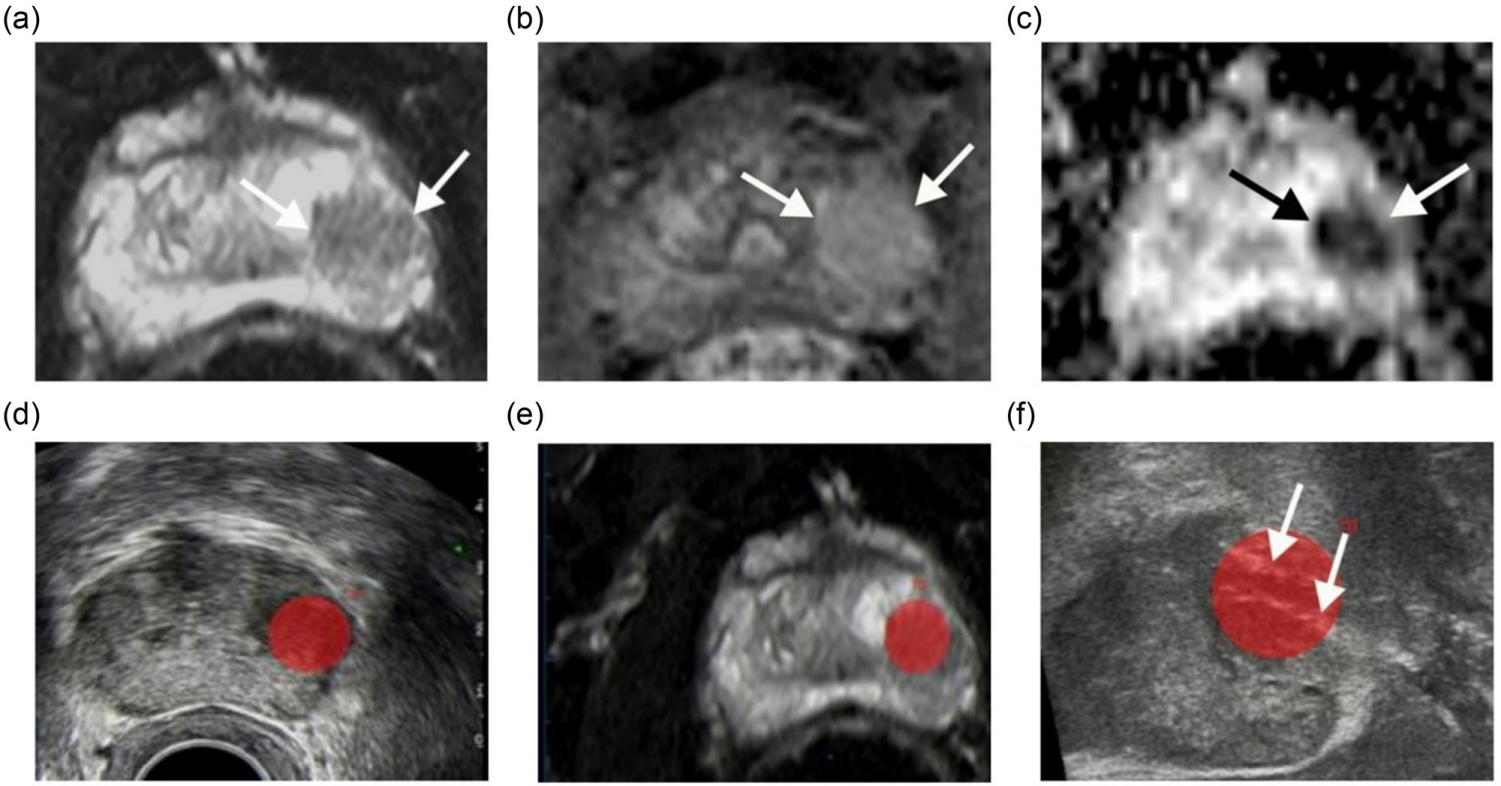
Fusion imaging and biopsy. A lesion was shown on mpMRI. Example of a 74 year old male (PSA 17.1 ng/mL) with a PI-RADS 5 lesion at 4 o’clock (white arrows) covered PZ and TZ: (a)–(c) T2, DCE, and ADC of a solid (arrowhead) are seen; (d) and (e) DWI image was uploaded on the ultrasound machine and coupling for MRI–TRUS fusion biopsy; the lesion was not obvious on the ultrasound images; (f) two biopsy needle marks were seen in target area (arrowhead).

The MRI–transrectal ultrasound (TRUS) fusion procedure is conducted by operators who possess expertise in MRI imaging. The process entails the transfer of prostate MRI data to compact disc and its subsequent integration into an ultrasound device. Subsequently, MRI volume alignment is performed by importing a minimum of two sequences of MRI data, typically T2WI and apparent diffusion coefficient (ADC), and utilizing MR/US fusion software to align the sagittal plane of the prostate MRI with a distinct cross-sectional plane showcasing the lesion. The real-time fusion of MRI and ultrasound images is employed to display the sagittal position of the prostate MRI and to manually adjust the ultrasound image for optimal alignment. T2 or ADC sequences are referenced to highlight lesions, identify regions of interest (ROI) that warrant further investigation, and mark lesions with red circles. Subsequently, the ultrasound probe should be repositioned to the sagittal plane in order to guide the puncture of the ROI, following the complete synchronization of MRI and ultrasound imaging. It is recommended that each lesion be punctured two to four times to ensure adequate coverage of the target area, as illustrated in Figure 2. Subsequently, a systematic transperineal biopsy comprising 12 cores should be conducted. Samples should be collected continuously and numbered for subsequent pathological examination.

### 2.3 TB with unilateral SB

**Figure 3:**
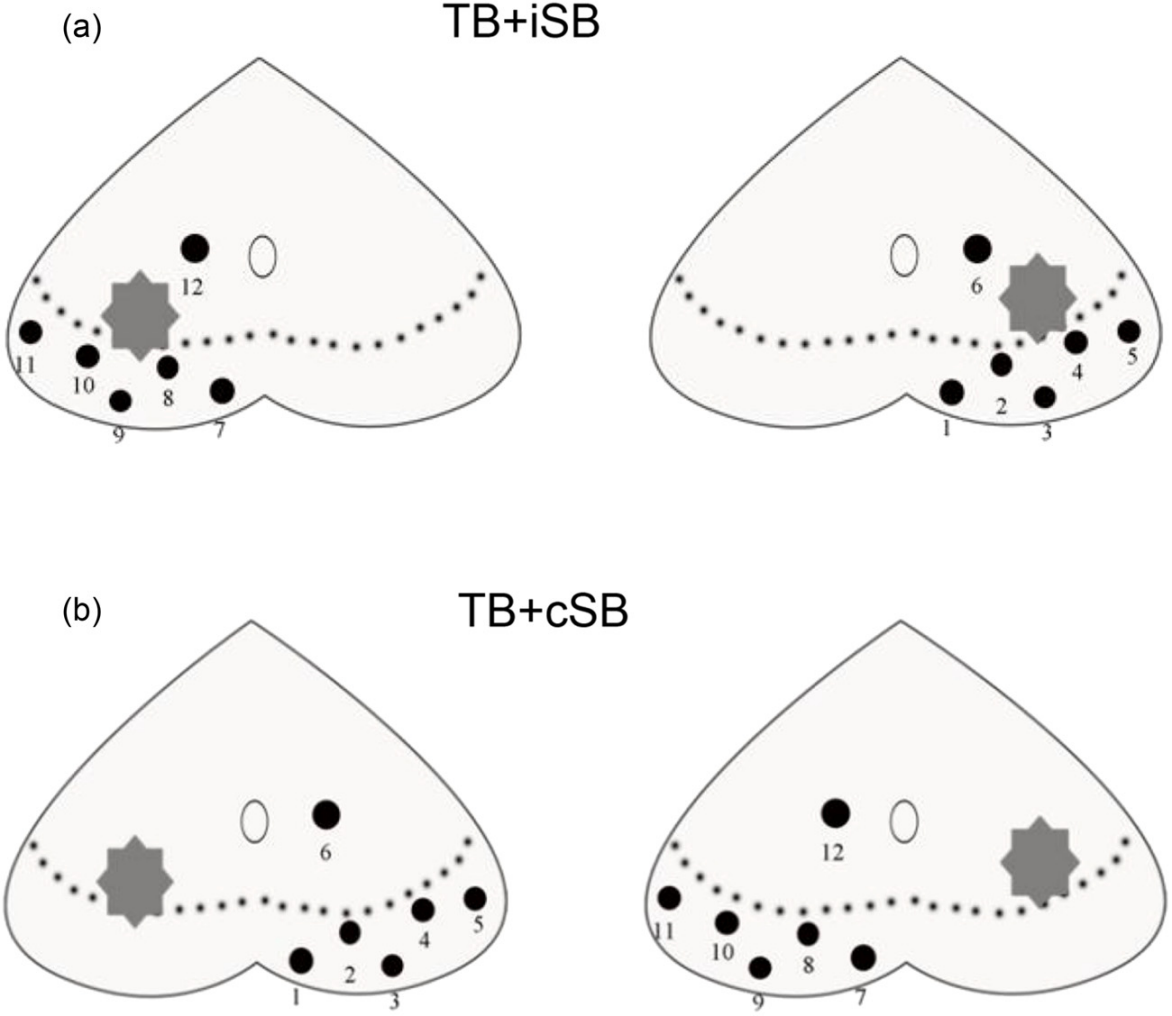
TB with unilateral SB: (a) in the TB + iSB group, two to four targeted biopsies were conducted on suspicious lesions, accompanied by six targeted systemic biopsies on the ipsilateral prostate tissue, and (b) in the TB + cSB group, two to four targeted punctures were performed on suspicious lesions, along with six systematic biopsies on the contralateral prostate tissue.

The study proposes two novel biopsy techniques: The targeted ipsilateral systemic biopsy (TB + iSB) technique involves a TB and a unilateral systemic biopsy on the same side of the target lesion. The targeted contralateral systemic biopsy (TB + contralateral system biopsy [cSB]) technique, on the other hand, involves a TB and unilateral systemic biopsy on the contralateral side of the target lesion (Figure 3).

### 2.4 Pathological analysis

Each punctured tissue sample is subjected to a series of processing steps, including fixation in a 10% formaldehyde solution, dehydration, embedding in paraffin, and sectioning. Subsequently, an independent analysis is conducted by one of two urology pathologists. The classification of each case is based on the primary and secondary Gleason scores, as well as the number and percentage of affected cores. A clinically significant disease is defined as one with a Gleason score of 3 + 4 or higher.

### 2.5 Statistical analysis

This study uses the results of combined biopsy (TB + SB) as the reference standard; this study aims to compare the accuracy and sensitivity of TB + iSB, TB + cSB, and TB in detecting csPCa using a two-test approach. Furthermore, the study will evaluate the discrepancies in positive needle count rates between TB + iSB, TB + cSB, TB + SB, and TB. The statistical significance of the observed differences will be determined at a significance level of *P* < 0.05. The data will be analyzed using the statistical software SPSS (Version 25.0, IBM Corporation, Chicago, IL, USA).

## 3 Result

### 3.1 Clinical and imaging features of prostate lesions

The demographic and tumor characteristics of the study sample are presented in Table 1. The mean age of the 222 patients was 70.42 ± 7.89 years, with a PSA range of 10.85 (6.86–17.71) ng/mL, a prostate volume range of 26.72 (30.48–57.20) mL, and a PSAD range of 0.27 (0.16–0.43) ng/mL. A total of 65.8% (146/222) of the needle biopsies of fusion lesions were conducted using two needles, 12.6% (28/222) used three needles, and 21.6% (48/222) used four needles. The lesions were identified in the right lobe in 52.3% of cases and in the left lobe in 47.3% of cases. The initial biopsy was conducted in 94.6% (210/222) of cases, while subsequent biopsies accounted for 5.4%. In accordance with the PI-RADS classification system, lesions classified as grade 3–5 were identified in 38.7% (86/222), 41.0% (91/222), and 20.3% (45/222) of cases, respectively. The overall detection rate of csPCa was 56.8% (126/222), which included 119 cases of adenocarcinoma, two cases of signet-ring cell carcinoma, four cases of mucinous carcinoma, and one case of signet-ring carcinoma with mucinous carcinoma. The biopsy results showed that 81 cases exhibited benign lesions, namely, five cases of prostatic intraepithelial neoplasia (PIN), one case of atypical small acinar proliferation (ASAP), one case of low-grade PIN (LG PIN), three cases of nonspecific granuloma (NG), and 71 cases of benign prostatic hyperplasia (BPH) with chronic interstitial inflammation.

**Table 1:** Clinical and imaging features of prostate lesions

Basic characteristics of patients and lesions	
**Characteristic**	
Patients (*n*)	222
Age (years), (mean ± sd)	70.42 ± 7.89 (45–84)
PSA (ng/mL), (median, IQR)	10.85 (6.86–17.71)
Prostate volume (mL), (median, IQR)	26.72 (30.48–57.20)
PSAD (ng/mL), (median, IQR)	0.27 (0.16–0.43)
Number of cores taken per location (median, IQR)	2 (2–4)
2, *n* (%)	65.8 (146/222)
3, *n* (%)	12.6 (28/222)
4, *n* (%)	21.6 (48/222)
**Lesion location**	
Left	47.3 (105/222)
Right	52.3 (117/222)
Biopsy type, *n* (%)	
Initial	94.6 (210/222)
Repeated	5.4 (12/222)
All PI-RADS score, *n* (%)	
3 (%)	38.7 (86/222)
4 (%)	41.0 (91/222)
5 (%)	20.3 (45/222)

### 3.2 Number of detected prostate cancers in PI-RADS 3–5 scores by various biopsy methods

For details regarding the incidence of PCa and csPCa in PI-RADS categories 3–5 (Table 2), the rates of detection for TB combined with SB in PI-RADS categories 3–5 were 17.44% (15/86), 73.62% (67/91), and 97.78% (44/45), respectively. When the combined biopsy results are considered as the reference standard, the detection rates of TB with iSB and cSB for csPCa in categories 3–5 were 100% (15/15), 98.51% (66/67), and 100% (44/44), respectively. In the 3th and 5th categories, TB with iSB successfully identified all csPCa cases, whereas in the 4th category, one csPCa case was not detected. Additionally, one csPCa case was not identified in the TB with cSB group in the 4th category.

**Table 2:** Number of detected prostate cancers in PI-RADS 3–5 scores by various biopsy methods

PI-RADS	TB (*n*, % of TB + SB)	SB (*n*, % of TB + SB)	TB + SB1 (*n*, % of TB + SB)	TB + SB2 (*n*, % of TB + SB)	TB + SB (*n*)
**3 = 86**					
Gleason score ≥ 6	15 (83)	11 (61)	18 (100)	15 (83)	18
Gleason score ≥ 7	11 (73)	10 (67)	15 (100)	11 (73)	15
**4 = 91**					
Gleason score ≥ 6	76 (96)	60 (76)	77 (97)	78 (99)	79
Gleason score ≥ 7	65 (97)	43 (64)	66 (99)	66 (99)	67
**5 = 45**					
Gleason score ≥ 6	44 (100)	38 (86)	44 (100)	44 (100)	44
Gleason score ≥ 7	43 (98)	35 (80)	44 (100)	43 (98)	44

Note: The table shows the number of prostate cancer (PCa) and significant cancer (csPCa) detected by PI-RADS 3–5 and the percentage in the total number of TB + SB detected by each puncture protocol.

### 3.3 Comparison of the accuracy, sensitivity, and negative predictive value (NPV) of prostate cancer diagnosis with a score of 3–5

Table 3 presents a comparison of the accuracy, sensitivity, and NPV of prostate cancer diagnosis with a score of 3–5. The diagnostic accuracy, sensitivity, and NPV of TB + iSB, TB + cSB, and TB for csPCa are superior to those of SB. In particular, the diagnostic accuracy of TB + iSB is markedly superior to that of TB + csPCa and TB (100% vs 95.35%, 95.35%), with no statistically significant difference compared to that of TB + SB (*P* = 1). Furthermore, the sensitivity and NPV of TB + iSB for csPCa exceed those of TB + cSB and TB (100% vs 73.33%, 73.33%). In addition, PI-RADS 4–5 groups TB, TB + iSB, and TB + cSB demonstrate comparable detection accuracy for csPCa.

**Table 3:** Comparison of the accuracy, sensitivity, and NPV of prostate cancer diagnosis with a score of 3–5

PI-RADS	Method	csPCa (Gleason score ≥ 7)				NPV
		Accuracy (95% CI)	*P*	Sensitivity (95% CI)	*P*	
3	TB	95.35 (88.52–98.72)	0.041	11/15 73.33 (44.90–92.21)	<0.001	94.67 (88.46–97.62)
	SB	94.19 (86.95–98.09)	0.021	10/15 66.67 (38.38–88.18%)	<0.001	93.42 (87.41–96.67)
	TB + iSB	1 (95.80–100)	1	1 (78.20–100)	>0.99	1
	TB + cSB	95.35 (88.52–98.72)	0.041	11/15 73.33 (44.90–92.21)	<0.001	94.67 (88.46–97.62)
4	TB	97.80 (92.29–99.73)	0.153	65/67 97.01 (89.63–99.64)	0.094	92.31 (75.40–97.92)
	SB	73.63 (63.35–82.31)	<0.001	43/67 64.18 (51.53–75.53)	<0.001	50.00 (42.06–57.94)
	TB + iSB	98.90 (94.03–99.97)	0.314	66/67 98.51 (91.96–99.96)	0.24	96.00 (77.43–99.41)
	TB + cSB	98.90 (94.03–99.97)	0.314	66/67 98.51 (91.96–99.96)	0.24	96.00 (77.43–99.41)
5	TB	97.78 (88.23–99.94)	0.312	43/44 97.72 (87.98–99.94)	0.306	50.00 (12.59-87.41)
	SB	80.00 (65.40-90.42)	0.001	35/44 79.55 (64.70-90.20)	<0.001	10.00 (5.84-16.60)
	TB + iSB	1 (92.13–100)	1	1 (91.96–100)	>0.99	1
	TB + cSB	97.78 (88.23–99.94)	0.312	43/44 97.72 (87.98–99.94)	0.306	50.00 (12.59–87.41)

The diagnostic sensitivity of TB + iSB and TB + cSB for csPCa is consistent with the NPV, with TB + iSB demonstrating higher values compared to TB (98.51% vs 97.01%) and TB + cSB (96.00% vs 92.31%). Furthermore, the sensitivity and NPV of TB + iSB for csPCa were also found to be superior to those of TB + cSB and TB (100% vs 97.72%, 97.72%; 100% vs 50%, 50%). TB + iSB exhibited the highest diagnostic accuracy, thereby reducing the likelihood of missed or incorrect diagnoses in comparison with TB + cSB and TB.

### 3.4 Comparison of positive needle count rate

Table 4 shows the comparison of positive needle count rates for TB, TB + iSB, TB + cSB, and TB + SB, with respective rates for csPCa.

**Table 4:** Comparison of positive needle count rates for PI-RADS 3-5 score prostate cancer diagnosis using various puncture biopsy methods

PI-RADS		csPCa (GS ≥ 7)		
		Positive needle rate (%)	*P*	Negative/positive
	TB	6.57		14.07
	SB	3.21	0.018	29.91
3	TB + iSB	4.08	0.127^#^	22.97
	TB + cSB	2.90	0.012	33.14
	TB + SB	3.75	0.056^#^	25.39
	TB	44.21		1.03
	SB	17.04	0.0000	4.44
4	TB + iSB	27.77	0.0000	2.16
	TB + cSB	20.47	0.0000	3.56
	TB + SB	21.91	0.0000	3.21
	TB	70.87		0.34
	SB	34.06	0.0000	1.77
5	TB + iSB	57.07	0.0000	0.67
	TB + cSB	34.24	0.0060	1.74
	TB + SB	40.94	0.0000	1.31

Note: TB has the highest positive needle rate. ^#^Indicating no statistical difference compared to TB. The ratio of negative to positive needles indicates the number of negative needles for each positive needle.

The highest incidence of positive acupuncture was observed in lesions with 3–5 points, which were identified as TB. The rate of positive needle counts for TB + iSB in the PI-RADS 3 group was found to be significantly higher than that of TB + cSB (4.08% > 2.90%), although no statistical significance was observed when compared to TB (*P* = 0.127, *P* = 0.056). In contrast, the positive needle count rates for TB + iSB, TB + cSB, and TB + SB in the PI-RADS 4–5 group were found to be lower than that of TB, with statistically significant differences observed (*P* < 0.05).

## 4 Discussion

Recent research has indicated that a combined biopsy is more effective than a targeted or systematic biopsy in the detection of csPCa. However, an elevated frequency of combined biopsies may potentially lead to an increased incidence of adverse events. The optimal methodology for biopsy planning remains a topic of debate within the academic community. In light of the limitations of systematic biopsy in identifying csPCa and its integration into combined biopsy protocols, a logical strategy for improving systematic biopsy techniques could offer a promising solution.

Our research demonstrates that when the pathological results of combined biopsy are used as the reference standard, TB + iSB exhibits the highest detection efficiency for csPCa in unilateral MRI lesions. Specifically, for the three lesion types examined, the detection rate of csPCa was found to be comparable to that of combined biopsy (100%, *P* = 1), with the positive needle count rate being only slightly lower than TB, showing no statistically significant difference (4.08% vs 6.57%, *P* = 0.127). Furthermore, the detection rate of csPCa in 4–5 lesion types using TB + iSB, TB + cSB, and TB is comparable to that of combined biopsy, with no statistically significant difference observed (*P* = 0.314, *P* = 0.314, *P* = 0.153; *P* = 1, *P* = 0.312, *P* = 0.312). Among all biopsy protocols, TB exhibits the highest positive needle count rate.

A number of factors may influence the diagnostic precision of csPCa across three lesion types, namely, the composition of biopsy specimens, the quality of MRI imaging, and the expertise of radiologists. In the initial biopsy cohort, the mean detection rate for the three lesions is 20% [10,11,12,13,14,15], while in the repeat biopsy cohort, the average rate is approximately 33% [16,17,18]. These findings are consistent with our own outcomes (20.93%, 18/86). The imaging characteristics of the three types of lesions are less distinct compared to the 4–5 types, which may allow for the concealment of cancer that may be missed by MRI. These lesions are intricately linked to their histopathological and molecular attributes, often displaying loose cell and vascular arrangements that present a challenge in differentiating them from the surrounding stromal background [19,20,21]. In Gleason grade 4, lesions with non-reticular main structures present additional challenges to the detection via MRI [22]. A research investigation into the tumor microenvironment within the low-risk subgroup of international society of urological pathology level 2 revealed that the presence of an invasive tumor microenvironment and elevated tumor mutation density may contribute to misdiagnoses in MRI imaging [23,24]. Additionally, the challenging nature of interpreting prostate MRI results represents a significant obstacle to accurately detecting cancer, with the proficiency of physicians in interpreting prostate MRI scans being of crucial importance in the identification of lesions [25]. The pathological findings indicate that prostatitis, particularly granulomatous prostatitis, may present a challenge in differentiating MRI pulse sequence signals from those of prostate cancer, potentially resulting in elevated false-positive rates [26].

Prior research has indicated that malignant tumors located outside the anticipated biopsy area are more frequently situated within the glandular tissue on the same side as the target lesion. The TB + iSB method demonstrated superior sensitivity and NPV in this study, suggesting a reduction in misdiagnosis and missed diagnostic rates for detecting csPCA.

Previous research has demonstrated the efficacy of fusion TB as a standalone approach for high-risk patients undergoing initial biopsy [9]. Lesions classified as PI-RADS 5 exhibit a specificity of nearly 90% in identifying prostate cancer [27]. The findings of our study suggest that the diagnostic rates of TB for PI-RADS 4–5 lesions in csPCa are 71.43% (65/91) and 95.56% (43/45), respectively. These rates are comparable to those of combined biopsy methods, exhibiting no statistically significant variance. Furthermore, the highest positive needle count rate was observed. Although the detection rate of TB for csPCa is comparable to that of combined biopsy, the heterogeneity of the disease and variations in lesion size between MRI and histological results [26] suggest that additional systematic biopsy may be necessary to identify csPCa that may have been missed by TB [12,18]. In our study, among the cases of csPCa detected through combined biopsy, two out of four types of lesions were identified by SB, representing 2.99% (2/67) of cases. One lesion was detected by cSB in the vicinity of the central axis, while the others were identified by iSB. One case, not within the TB category, was overlooked within the five categories, representing 2.27% (1/44) of cases, and was only discovered through iSB. Overall, the diagnostic accuracy of TB for detecting 4–5 types of lesions in csPCa is somewhat inadequate as a biopsy strategy. The potential benefits of reducing the number of biopsies while maintaining diagnostic accuracy warrant further investigation.

In the process of joint biopsy, samples are obtained from lesions that have been identified on MRI, as well as from the glandular lobular symmetry at random. The number of targeted biopsies performed is a significant determinant of the positivity rate. It is typically recommended to administer a minimum of two injections, with five injections being regarded as the optimal number for the detection of csPCa and the minimization of complications associated with an excessive quantity of contrast agent [28]. The efficacy of symmetric SB in detecting csPCA for the purpose of preventing missed diagnoses of TB may be variable. In the examination of regional TB, both TB and expanded biopsy were conducted on glands located within a 2 cm radius of the target lesion edge. The findings indicated that both TB and combined biopsy yielded the most optimal detection outcomes [5]. The spatial configuration of biopsy needles in our study model is analogous to that of regional TB. Nevertheless, we devised a model comprising a single visible lesion, with the objective of preventing potential cross-contamination and ensuring unbiased detection rates for multiple lesions. The positive needle count rate of TB + iSB is higher than that of TB + cSB, indicating that SB on the same side of the lesion is more likely to detect a missed diagnosis of TB in csPCA.

It should be noted that the research presented here is not without limitations. It is important to note that this study employed a retrospective design, which may have introduced a degree of selective bias. In addition, PSA density was not employed for the stratification of csPCA risk, and MRI demonstrated discrepancies in the negative predictive value of csPCA. Furthermore, the diagnosis of csPCa presence was not based solely on the results of the initial histological examination. The combined biopsy method yielded the lowest rate of csPCa upgrading. Moreover, this study did not take into account factors such as prostate size, lesion size, and the absence of a template prostate biopsy as a reference standard was not taken into consideration in this study.

## 5 Conclusion

The findings of our study revealed that the combination of TB + iSB as a biopsy method was an effective approach for the detection of csPCa, as evidenced by the favorable statistical outcomes. The diagnostic performance of csPCa across three lesion types is equivalent to that of combined biopsy, while also reducing the number of required punctures. Moreover, the detection rate of csPCa in lesions scoring 4–5 in TB is comparable to that of combined biopsy, with the fewest punctures required. These findings suggested the potential for further refinement of biopsy techniques. Further large-scale prospective studies are necessary to validate the clinical effectiveness of this method.

## Abbreviations

TB: targeted biopsy
uSB: unilateral systematic biopsy
csPCa: clinically significant prostate cancer
MRI: magnetic resonance imaging
SB: standard biopsy
PI-RADS: Prostate Imaging Reporting and Data System
mpMRI: multiparametric magnetic resonance imaging
ROI: regions of interest
PIN: prostatic intraepithelial neoplasia
ASAP: atypical small acinar proliferation
LG PIN: low-grade PIN
NG: nonspecific granuloma
BPH: benign prostatic hyperplasia
cSB: contralateral system biopsy
NPV: negative predictive value
